# Longitudinal curriculum development: gradual optimization of a biochemistry seminar

**DOI:** 10.3205/zma001281

**Published:** 2019-11-15

**Authors:** Achim Schneider, Michael Kühl, Susanne J. Kühl

**Affiliations:** 1 Ulm University, Institute for Biochemistry and Molecular Biology, Ulm, Germany

**Keywords:** curriculum development, course development, vertical integration, competence orientation, inverted classroom, flipped classroom, biochemistry, quality management

## Abstract

**Objective: **The Master Plan for Medical Studies 2020 places additional emphasis on curricular development processes. In addition, institutes may recognize a need to optimize their courses, for example because of poor evaluations. Frequently, however, the resources required for comprehensive optimizations are not available. In the present study, we aimed to use the example of a biochemistry seminar that takes place in the preclinical part of the medical degree at Ulm University Medical School to show how a course can be successfully optimized in small steps and evaluation results can be used for quality assurance.

**Methods: **Similar to a continuous improvement process (CIP), over the course of five years a biochemistry seminar was gradually optimized in three steps. This process used structural, methodological, and content components, such as vertical integration, the inverted classroom method, and competence orientation. For quality assurance, we analyzed the official, standardized evaluation sheets from a total of n=1248 students. We evaluated the optimization process on the basis of

1. responses to evaluation statements that were arranged into categories such as “organization, structure, implementation” and “quality of teaching,”
2. free text information, and
3. the results of a pilot study conducted in parallel.

We then determined the usefulness of the students’ evaluation results for evaluating the optimization process.

**Results:** Each developmental step resulted in a significantly more positive overall evaluation of the seminar by the students. This result was independent of whether the development was on a structural or methodological/content-related level. In addition, the evaluations of the categories that were optimized were significantly better. For example, the vertical integration and introduction of the inverted classroom method were accompanied by a better evaluation of the “quality of teaching” and a change in the structure led to a higher score in the category “organization, structure, implementation.” A comparison with the free text evaluation sheets and the results of the pilot study supports the results.

**Conclusion: **Although optimization of a curriculum or course is a major task, it can also be successfully completed in small steps. With this approach, new learning goals, for example as required in the Master Plan for Medical Studies 2020, can be continuously integrated and student satisfaction with a course can be increased. Student evaluation results can represent a kind of quality assurance in this process and can provide important impulses for optimization.

## Zusammenfassung

**Zielsetzung: **Der Masterplan Medizinstudium 2020 rückt curriculare Entwicklungsprozesse weiter in den Vordergrund. Aber auch institutsintern kann es Gründe wie z.B. schlecht ausfallende Evaluationen geben, die eine Optimierung von Lehrveranstaltungen nahelegen. Häufig stehen jedoch für umfassende Weiterentwicklungen nicht die notwendigen Ressourcen bereit. Mit der vorliegenden Studie wollen wir anhand eines Biochemie-Seminars des vorklinischen Studienabschnitts des Regelstudiengangs der Humanmedizin in Ulm exemplarisch zeigen, wie die Optimierung einer Lehrveranstaltung in kleinen Schritten gelingen kann und Evaluationsergebnisse zur Qualitätssicherung herangezogen werden können.

**Methodik: **In Anlehnung an einen kontinuierlichen Verbesserungsprozess (KVP) wurde ein Biochemie-Seminar über fünf Jahre hinweg in drei Zyklen Schritt für Schritt weiterentwickelt. Dabei wurden strukturelle, methodische und inhaltliche Komponenten, wie vertikale Integration, die Inverted Classroom-Methode und Kompetenzorientierung eingesetzt. Zur Qualitätssicherung wurden die offiziellen und standardisierten Evaluationsbögen von insgesamt N=1248 Studierenden herangezogen. Anhand einzelner Evaluationsfragen, die in verschiedene Kategorien wie „Organisation, Struktur, Aufbau“ oder „Didaktische Umsetzung“ gegliedert sind, Freitextangaben sowie anhand von Ergebnissen einer parallel durchgeführten Pilotstudie wird die Weiterentwicklung differenziert bewertet und die Nützlichkeit von Evaluationsergebnissen herausgearbeitet.

**Ergebnisse:** Jeder Entwicklungsschritt führte auf Studierendenseite zu einer signifikant positiveren Gesamtbewertung des Seminars. Dieses Ergebnis ist unabhängig davon, ob die Entwicklung auf struktureller oder methodisch-inhaltlicher Ebene stattfand. Darüber hinaus finden sich signifikant bessere Bewertungen derjenigen Kategorien, die weiterentwickelt wurden. So geht beispielsweise die vertikale Integration sowie die Einführung der Inverted Classroom-Methode mit einer besseren Bewertung der „Didaktischen Umsetzung“ einher und die strukturelle Veränderung führte schließlich zu einer Verbesserung der Kategorie „Organisation, Struktur, Aufbau“. Ein Abgleich mit den Freitexten der Evaluationsbögen sowie mit den Ergebnissen der Pilotstudie unterstützt die Ergebnisse.

**Schlussfolgerung: **Wenngleich die Weiterentwicklung eines Curriculums oder einer Lehrveranstaltung eine große Aufgabe ist, so kann diese auch erfolgreich in kleinen Schritten erfolgen. Auf diesem Weg ist es möglich, kontinuierlich neue Lernziele, wie z.B. im Masterplan Medizinstudium 2020 gefordert, zu integrieren, und die Zufriedenheit der Studierenden bezüglich einer Lehrveranstaltung zu erhöhen. Studentische Evaluationsergebnisse können in diesem Prozess als eine Art der Qualitätssicherung genutzt werden und wichtige Impulse zur Weiterentwicklung geben.

## 1. Introduction

Efforts are being made on both the national and international level to optimize medical education (see [1], [2], [3], [4], [http://www.nklm.de]). The Federal Government of Germany also wants to redesign medical education and, within the framework of its coalition agreement [3], is pursuing the implementation of the “Master Plan for Medical Studies 2020,” (MM2020), which was adopted in 2017 [4]. The MM2020 calls for more practical relevance by linking clinical and theoretical content, teaching communication skills, and testing and continuously improving competence-oriented teaching formats.

The German Medical Faculty Association created the National Competence-based Catalogue of Learning Objectives in Medicine [http://www.nklm.de] with the aim of facilitating the optimization of their curricula and, at the same time, creating a uniform basis. The catalogue includes learning objectives regarding both the roles of physicians as medical experts, communicators, and team members and physician-patient interactions, among other things.

Institutes may also have their own reasons for optimizing a course, such as poor evaluation results, for example.

In view of the current developments, every faculty, institute, and person responsible for teaching is faced with the challenge of adapting the curriculum or their own courses to future requirements. There are several ways to do this, but as a rule not all adaptations can be implemented as a one-time innovation, for example because the necessary resources are not available or comprehensive changes entail too much risk.

One possibility to implement comprehensive developments, however, is to establish subgoals and realize them one by one, thus gradually moving closer to achieving the overall goal. This method of small steps is used in a similar form in quality management (continuous improvement process [CIP] [5]).

The advantage of using this approach when optimizing courses is that the first steps can be undertaken with limited resources, thus ensuring the capacity to act. It also makes it easier to identify and correct minor developments that do not produce the desired result. To structure the process, one can use models for curriculum development (e.g. Kern’s six-step approach [6]) or quality assurance (e.g. PDCA/PDSA cycle [7]) also within the small steps. Similarly, small steps can be planned and implemented in the sense of best evidence medical education (BEME) [8], [9].

### 1.1 Objective

To evaluate whether the method of small steps can be successfully applied to optimize a course, we implemented the method in a biochemistry seminar that takes place in the preclinical part of the medical degree at Ulm University Medical School.

The changes to the seminar that were implemented between the winter semester (WS) 2012/13 and summer semester (SS) 2017 were changing the scheduling of the seminar, vertical integration, changing the teaching concept, and integrating competence-oriented learning objectives. Apart from the change in scheduling of the seminar, these changes met some of the requirements of the MM2020.

We studied the following questions related to these changes:

1. Can a course be successfully optimized in small steps?
2. Are official evaluation measures collected as part of faculty quality management suitable for depicting an optimization in small steps?

## 2. Methods

### 2.1 Course to be optimized and starting position

The optimization in small steps was implemented in the “Integrated Seminar” at the Medical Faculty in Ulm, which – as stated Section 2, Paragraph 2 of the German Medical Licensing Regulations for Physicians – is a compulsory seminar on biochemistry for medical students in the pre-clinical part of their degree. Sixteen groups of 20 students were assigned to the biochemistry seminar in each year of the study period (2012 to 2017). Depending on the year, five to seven different experienced lecturers taught the seminar, whereby each group of students was led by only one lecturer (see [10] for details). During the study, a total of 11 different lecturers were responsible for teaching the seminar, five of them only occasionally: these five lecturers taught only 12 of the 96 seminar groups over the course of the study (in 2012, 2013, 2016, 2017), while the remaining 84 seminar groups were taught by the remaining six lecturers.

In the WS 2012/13 seminar, students gave presentations on the principles of biochemistry (e.g. DNA, transcription, translation, and proteins) and the respective lecturer moderated the presentations. The teaching focused on the learning of facts, was neither student nor competence oriented and was scheduled at the end of the 3rd semester, directly after a work-intensive course.

#### 2.2 Building blocks for optimizing the seminar

**Figure 1:**
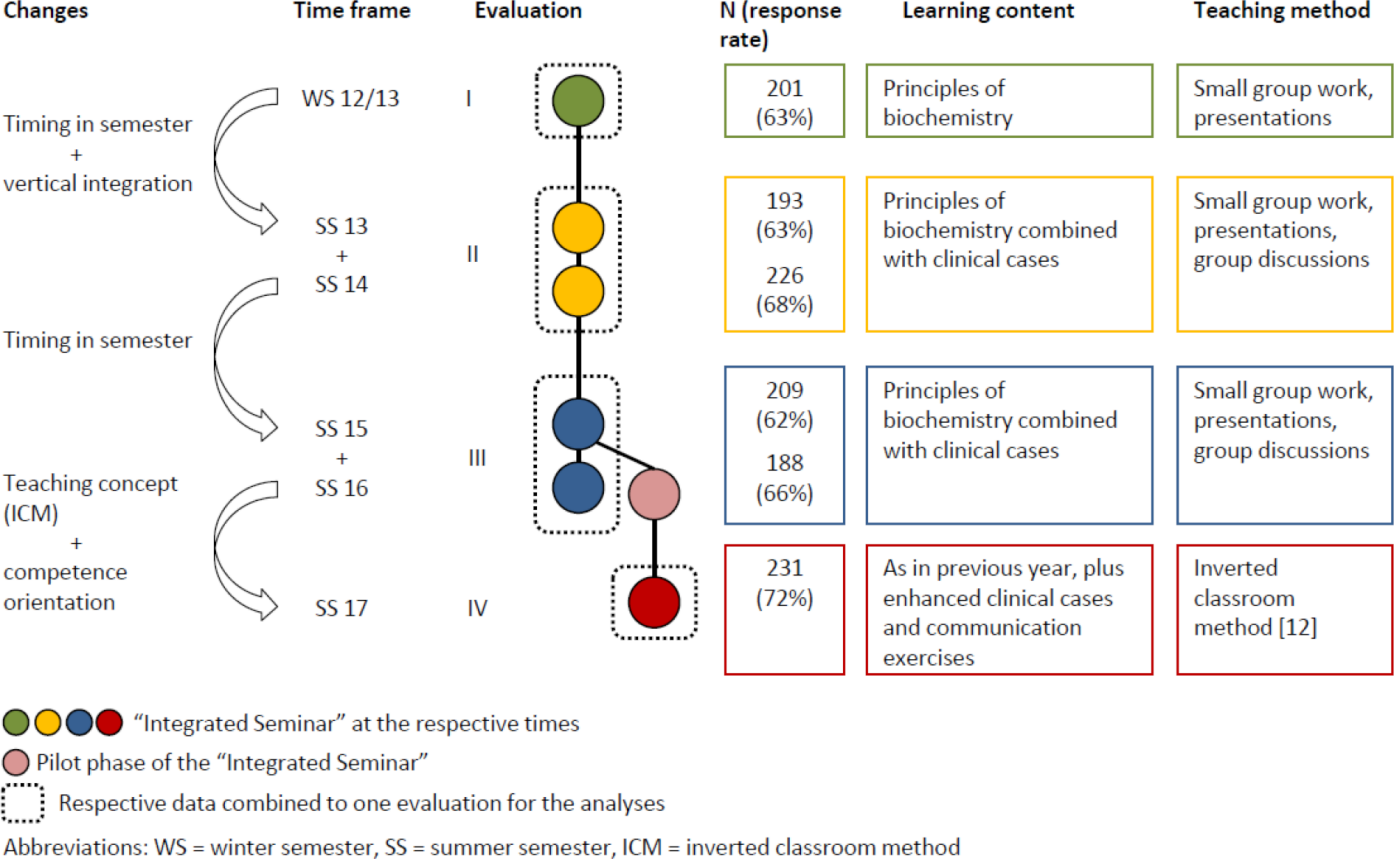
Schematic presentation of the changes to the curriculum between the winter semester 2012/2013 (WS 12/13) and the summer semester 2017 (SS 17). The sample for the analyses comprised 1248 medical students at the University of Ulm who participated in the seminar and completed an evaluation in the period WS 2012/13 to SS 2017.

Figure 1 (Fig. 1) illustrates the optimization of the seminar in the respective periods.

#### Structural changes

To better coordinate the curriculum (temporal proximity of lecture and seminar), in the SS 2013 the biochemistry seminar was moved from the end of the 3^rd^ semester to the end of the second semester. In SS 2015, it was moved again to the middle of the second semester.

#### Vertical integration

One way to increase the practical relevance of the preclinical part of the degree is to include clinical content, an approach that is also referred to as “vertical integration”. Besides increasing the vividness of basic science content and in turn its relevance, vertical integration supports the development of students’ clinical decision-making competence [11], [12].

In the SS 2013, the principles of biochemistry were taught on the basis of the clinical presentation of Osteogenesis imperfecta, Ehlers-Danlos syndrome, and scurvy to illustrate the relevance of the topics. This change also contributed to fulfilling the MM2020’s requirements for more practical relevance and learning objectives of the National Competence-based Catalogue of Learning Objectives in Medicine (e.g. the doctor as a medical expert, Learning goal 5.2.1.1). In SS 2017, the examples of disease presentations were enhanced (see [10] for details).

##### The inverted classroom method (ICM)

The ICM is a blended learning method in which students initially acquire factual knowledge on their own (self-learning phase). This creates space in the subsequent presence phase for students to apply, analyze and synthesize the learned content in the group and with the lecturer [13], [14]. According to the revised taxonomy of Bloom [15], this approach enables a higher cognitive learning process. The ICM can also be used for competence-oriented teaching [10] and to increase student motivation [16].

The ICM therefore allows the MM2020 requirements to be met for a competence-oriented design of medical education and for the testing and continuous improvement of corresponding teaching formats. The concrete design of the ICM used here was successfully piloted in the SS 2016 (see [10] for details) and was introduced for all seminar groups in SS 2017.

##### Communication skills

In recent decades, communication skills have become a key aspect of medical education. Studies and consensus papers on, ideas about, and models for integrating communicative skills into a curriculum have been published demonstrating the efficacy of communication skills training [17], [18], [19], [20], [21]. The teaching of communication skills is also a key element of MM2020.

After a successful pilot study in SS 2016 (see [10] for details), in the SS 2017 learning objectives from the National Competence-based Catalogue of Learning Objectives in Medicine regarding communication in a team, with colleagues and with laypeople were integrated into all seminar groups in the form of role-playing games and tasks for preparing oral examination scenarios.

#### 2.3 Data collection and analysis

For the analyses, we used data that had been collected voluntarily and anonymously from students on the official, standardized evaluation forms of the Medical Faculty Ulm for the periods WS 2012/13 to SS 2017. We analyzed the interval-scale data for the categories “organization, structure, implementation of the seminar,” “commitment to teaching of the lecturers,” and “quality of teaching in the seminar”; each of these categories consisted of three evaluation statements that were rated on a Likert-type scale ranging from 1=“does not apply at all” to 6=“fully applies” (see table 1 (Tab. 1)). In addition, we evaluated the overall evaluation of the seminar, which had been assessed on a Likert-type scale ranging from 1=“very good” to 6=“insufficient”, and the responses to the dichotomous question whether there was a “need for action to optimize the seminar.”

We also quantified free texts: As part of the evaluation, students can express praise in one free text field and criticism in another. Positive or negative free texts in the respective free text fields were counted and summed for each period of time. Negative free texts in the free text field for praise were not included and vice versa, and neutral free texts were not evaluated. Representative free texts are quoted in suitable places below, and more example free texts can be found in the attachment 1 .

Because there were no significant differences between the data from the years between which the seminar was not changed (see figure 1 (Fig. 1)), the respective data were pooled. Differences between the periods in which changes were made were tested by analyses of variance without repeated measurements, and individual values were compared with the post hoc test Tukey’s HSD. Changes in the “need for action” and the quantified free texts were analyzed by chi-square tests. Differences with a p-value < 0.05 were considered significant and relevant. All analyses were performed with IBM SPSS Statistics for Windows [22].

The Ethics Committee of the University of Ulm considered it unnecessary to submit an official application.

## 3. Results

### 3.1 Evaluations in WS 2012/13

**Figure 2:**
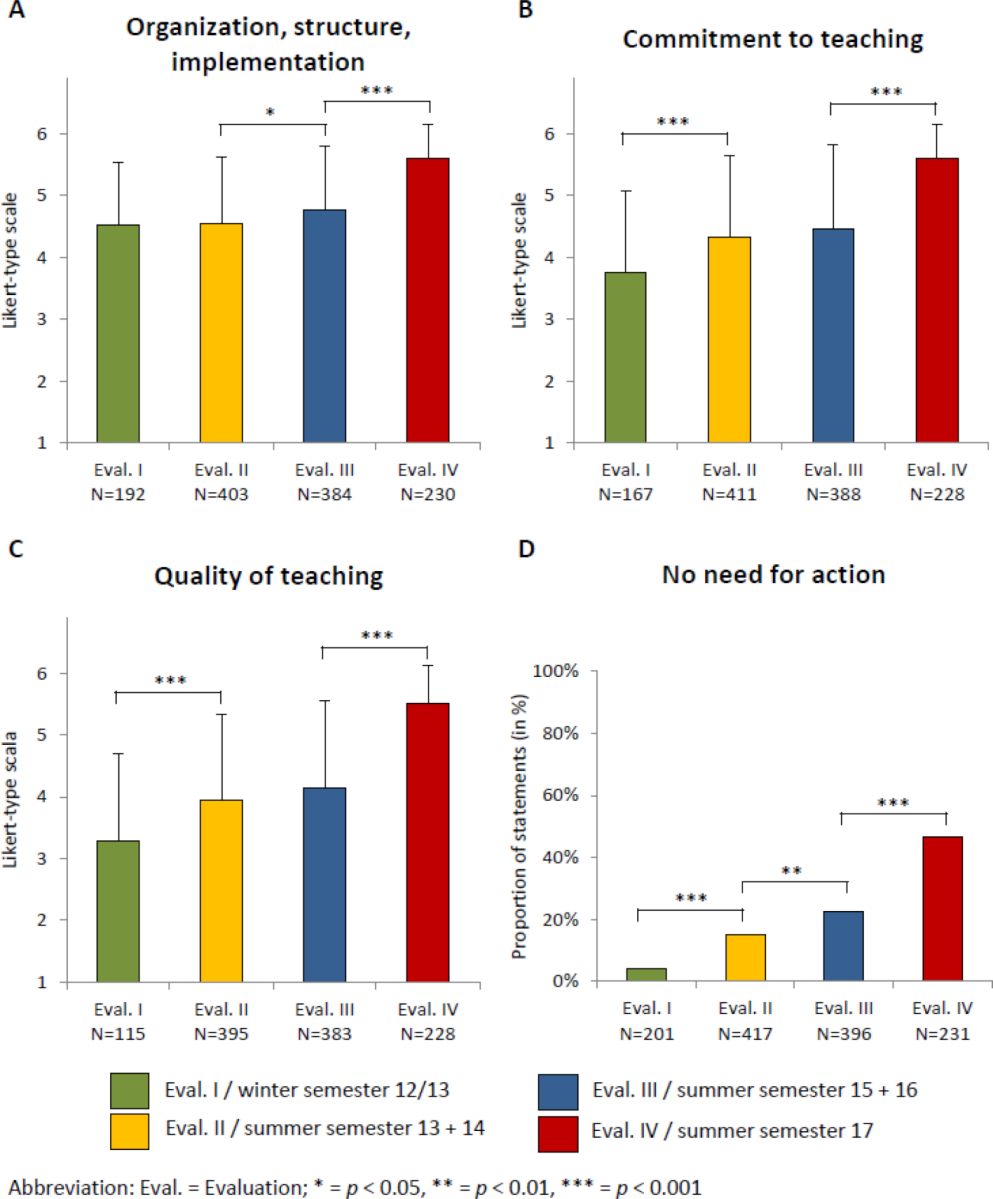
A-C: Mean scores of the students’ evaluations on the respective scale A: Organization, structure, implementation of the seminar; B: Commitment to teaching of the lecturers involved in the seminar; C: Quality of teaching in the seminar; Likert-type scale ranging from 1=“does not apply at all” to 6=“fully applies”. D: Proportion of students stating that there is no need for action regarding optimizing the seminar (in %).

**Figure 3:**
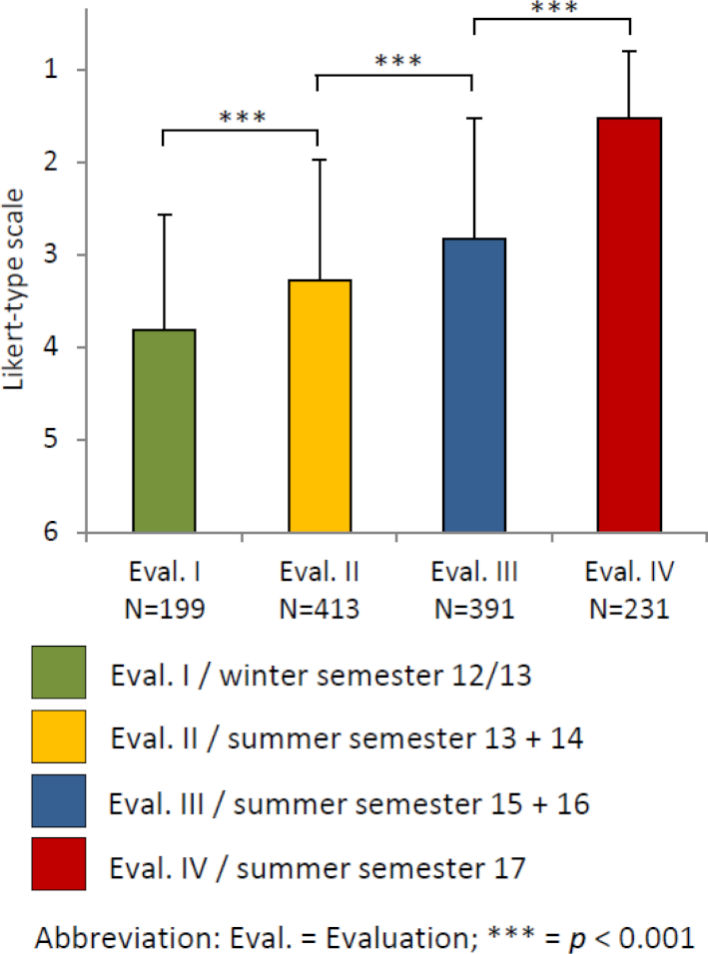
Mean overall scores of the students’ evaluation of the “Integrated Seminar” rated on a Likert-type scale ranging from 1=“very good” to 6=“insufficient”.

**Figure 4:**
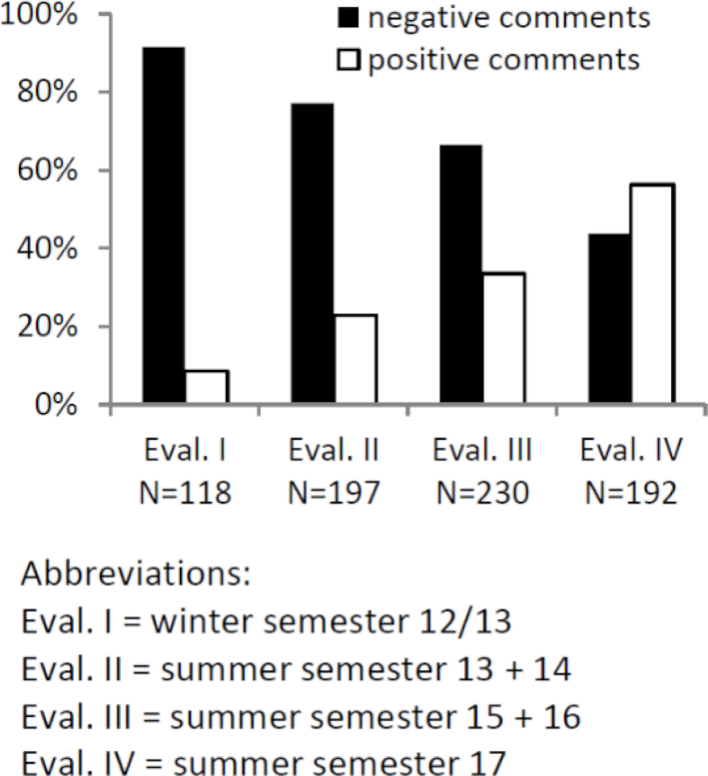
Frequency of positive and negative free text comments at each evaluation time point. All differences between evaluation time points are significant (p<0.05 for each).

The analysis of the evaluations in WS 2012/13 found that students often did not recognize the relevance of the basic topics (quote: “It might be better to link the presentations to certain diseases, to make the relevance clearer [...]”) and were dissatisfied with the communication of facts through presentations by their fellow students (quote: “20 presentations in a row is simply not effective [..]”). This is also reflected in the relatively low scores in the categories “commitment to teaching” and “quality of teaching,” as well as in the “overall evaluation” (see figure 2 (Fig. 2) and figure 3 (Fig. 3)). Because of the large number of presentations and the scheduling of the seminar at the end of the 3^rd^ semester, the motivation during and satisfaction with the seminar were low (quote: “At the end of the really exhausting 3^rd^ semester, it was just ‘get this over with, too ...’“). The majority of free texts (91.5%) were negative (see figure 4 (Fig. 4)).

#### 3.2 Evaluations in SS 2013 and 2014

Moving the seminar to the end of the 2^nd^ semester did not result in any significant difference in the area of “organization, structure, implementation” (p= 0.998; see figure 2 (Fig. 2), point A). Hints as to why not were apparent from the following free texts: “Why now? After the semester is ‘over’?”; “[...] then parallel to the psych course [...].”

On the other hand, after the vertical integration of clinical diseases the evaluations of “commitment to teaching” and “quality of teaching” improved significantly (see figure 2 (Fig. 2), points B and C), which was also expressed in some free texts, for example: “It was good that the lecturer tried to explain what we did not understand by using practical examples.” There was also a significant improvement in the “need for action” (see figure 2 (Fig. 2), point D) and the “overall evaluation” (see figure 3 (Fig. 3)). The proportion of negative free texts decreased to 77.2% (see figure 4 (Fig. 4)).

#### 3.3 Evaluations in SS 2015 and 2016

After moving the seminar to the middle of the second semester, the scores for “organization, structure, implementation” increased significantly (see figure 2 (Fig. 2), point A). There were also only a few critical free texts about the scheduling of the seminar and significant improvements in both the “need for action” (see figure 2 (Fig. 2), point D) and the “overall evaluation” (see figure 3 (Fig. 3)). The negative free texts decreased further to 66.5% (see figure 4 (Fig. 4)).

An analysis of the free texts found that there were still some criticisms:

1. Poor motivation and concentration (quote: “The topics are complicated, and it’s difficult to concentrate for four hours”);
2. poor learning atmosphere (quote: “[...] did not manage to create a positive learning atmosphere at any of the sessions [...]”);
3. unclear learning objectives (quote: “[...] because I could not see any relevance for exams or the like.”).

#### 3.4 Evaluations in SS 2017

The introduction of ICM and the communication-related learning objectives were associated with significant increases in the areas of “organization, structure, implementation,” “commitment to teaching,” and “quality of teaching” (see figure 2 (Fig. 2), points A-C). There were also significant improvements in the “need for action” and “overall evaluation” (see figure 2 (Fig. 2), point D and figure 3 (Fig. 3)). The proportion of negative free texts decreased to 43.8% (see figure 4 (Fig. 4)). The following free text is representative of frequent statements about the ICM: “I love this model! Should also be extended to other seminars! The time requirement is almost the same, but the learning is much more productive!”

## 4. Discussion

### 4.1 Baseline situation (WS 2012/2013)

The poor overall evaluation and the clear need for action in the evaluation of the WS 2012/13 formed the starting point for the optimization of the seminar. An analysis of the free texts revealed worthwhile steps that could be taken, illustrating the usefulness of student evaluations.

#### 4.2 First step (SS 2013 and 2014)

Moving the seminar to an earlier time in the semester did not affect the student evaluation of “organization, structure, implementation.” A good explanation for this finding is that the move resulted in the seminar competing with another course and that part of the problem, i.e. the scheduling at the end of the semester, had not been solved. Another explanation is related to the sensitivity of this category and is discussed in more detail below (section 4.3).

The improvements in the areas of “commitment to teaching” and “quality of teaching” and the declining proportion of critical free texts were at least partly due to the vertical integration of clinical aspects, which is apparent from the free texts. Studies have shown that vertical integration can have a positive effect on motivation and the depth of understanding [23], [24]. Lecturers may have been subconsciously sensitized to these issues through the change to the seminar, which in turn could have affected the evaluation results, particularly in the area of “commitment to teaching.” The respective proportion of these effects (vertical integration or sensitization) cannot be clarified on the basis of the available data. This also applies to the improvement of the overall evaluation, although this evaluation gives a positive general impression of the first step.

#### 4.3 Second step (SS 2015 and 2016)

The rather minor improvement in the area of “organization, structure, implementation” can be attributed to the fact that the evaluation statements on the category were based less on the scheduling of the seminar in the curriculum and more on the organizational aspects of the seminar (see table 1 (Tab. 1)). In the free texts, there was only occasional criticism about the scheduling, so we did not change it again. Taken together, the overall evaluation, the decrease in the need for action, and the proportion of critical free texts indicated that the change was positive.

#### 4.4 Third step (SS 2017)

After the introduction of the ICM and the associated structural change in the seminar, the evaluation in the area of “organization, structure, implementation” further improved significantly. This finding is in line with other studies, which also found that students have a positive perception of the organizational structure of ICM [13], [25].

The ICM was able to address most of the outstanding needs regarding methodological and teaching aspects. The pilot study of the ICM in the SS 2016 showed that the ICM and the associated involvement of other teaching elements can clarify the relevance of the learning content and increase motivation [10]. The finding in the present study that this effect was also reflected in the categories “commitment to teaching” and “quality of teaching” was also supported by the results of the pilot study: This controlled intervention study found positive changes in the underlying evaluation statements of these categories within the same cohorts, just as we did here between different cohorts [10]. These observations are consistent with the results of other studies, which described more intensive learning and increased motivation as a result of the ICM [16], [26]. Because both the seminar content and method were optimized, it is not possible to further differentiate between individual effects on “commitment to teaching” and “quality of teaching.”

Overall, the improvement in the overall evaluation, the decrease in the need for action and the increase in positive free texts demonstrate that the third step was successful.

#### 4.5 Method of small steps

In our opinion, the optimization of the biochemistry seminar in small steps was successful, even though each individual step, for example the first change in the scheduling of the seminar, did not directly result in an improvement. However, the associated reasons could be easily identified and, in the next step, eliminated, allowing an improvement to be achieved. The advantage of the method is apparent in the small-step approach, which allows relatively simple adjustments to be made. Evidence-based teaching methods can also be integrated into this approach and, if adapted to the local conditions, might lead to more or stronger positive effects.

#### 4.6 Suitability of student evaluations

The validity of student evaluations can be viewed critically [27]. Nevertheless, the data presented here show that student evaluations can be used to depict optimization of a course in small steps, at least to a limited extent. For example, the student evaluations of “organization, structure, implementation” did not change between WS 2012/13 and SS 2013, but they did between the SS 2014 and SS 2015. It is also necessary to draw attention to their limits, however. For example, we could only evaluate the SS 2017 results on “commitment to teaching” and “quality of teaching” in a more differentiated way in association with the results of the pilot study. In the same way, we could only largely rule out effects of the cohort by comparing the results of the pilot study, which was performed within one cohort, with those of the current study. The usefulness of evaluation results also depends on how differentiated the inquiry is, as the discussion on the second development step shows.

#### 4.7 Limitations

The present study focused on student evaluations. Thus, it could not describe the extent to which the optimization had an impact on learning performance. However, the evaluation of learning success on the basis of multiple choice questions in the pilot study indicated non-inferiority of the final step [10].

The WS 2012/13 sample showed a high degree of variability between some evaluation time points (see figure 2 (Fig. 2)), which may have had an impact on the results. We attribute this variability to the high dissatisfaction with the format of the seminar at that time, which may have led students to not respond to the statements on “quality of teaching.” 

A further limitation may be the change in lecturers over the seminar optimization period. Because only one eighth (12 of 96) of the groups were affected, the various lecturers had comparable qualifications, and the changes happened in several different years and were not made with the aim to improve the seminar, however, we do not think it likely that the changes had a significant impact on the results. On the other hand, the continuous improvement in the evaluation results, despite personnel changes, could also be interpreted in favor of a lecturer-independent optimization of the seminar.

## 5. Conclusion

In summary, the present study shows how a course can be continuously developed in small steps to the benefit of the students. It is thereby noteworthy that each optimization of the various aspects of a course, such as structural, content- and teaching-related, and methodological characteristics, can help to improve student satisfaction. Thus, different approaches can be used, depending on the local conditions.

Not every change has to result in a significant improvement. Nevertheless, the experiences and student evaluations can be used for further optimizations and thus small steps can together lead to significant improvements. This approach is particularly suitable when little time or few resources are available to implement major innovations. In this way, necessary and required developments, such as those of the MM2020, can be addressed in an evidence-based approach and medical students can thus be better prepared for the clinical phase of their education and future demands.

## Acknowledgement

We would like to thank the Dean of Studies of the Faculty of Medicine of the University of Ulm and the lecturers and students of the seminar.

## Competing interests

The authors declare that they have no competing interests. 

## Supplementary Material

Excerpts of critical free text (anonymized)

## Figures and Tables

**Table 1:**
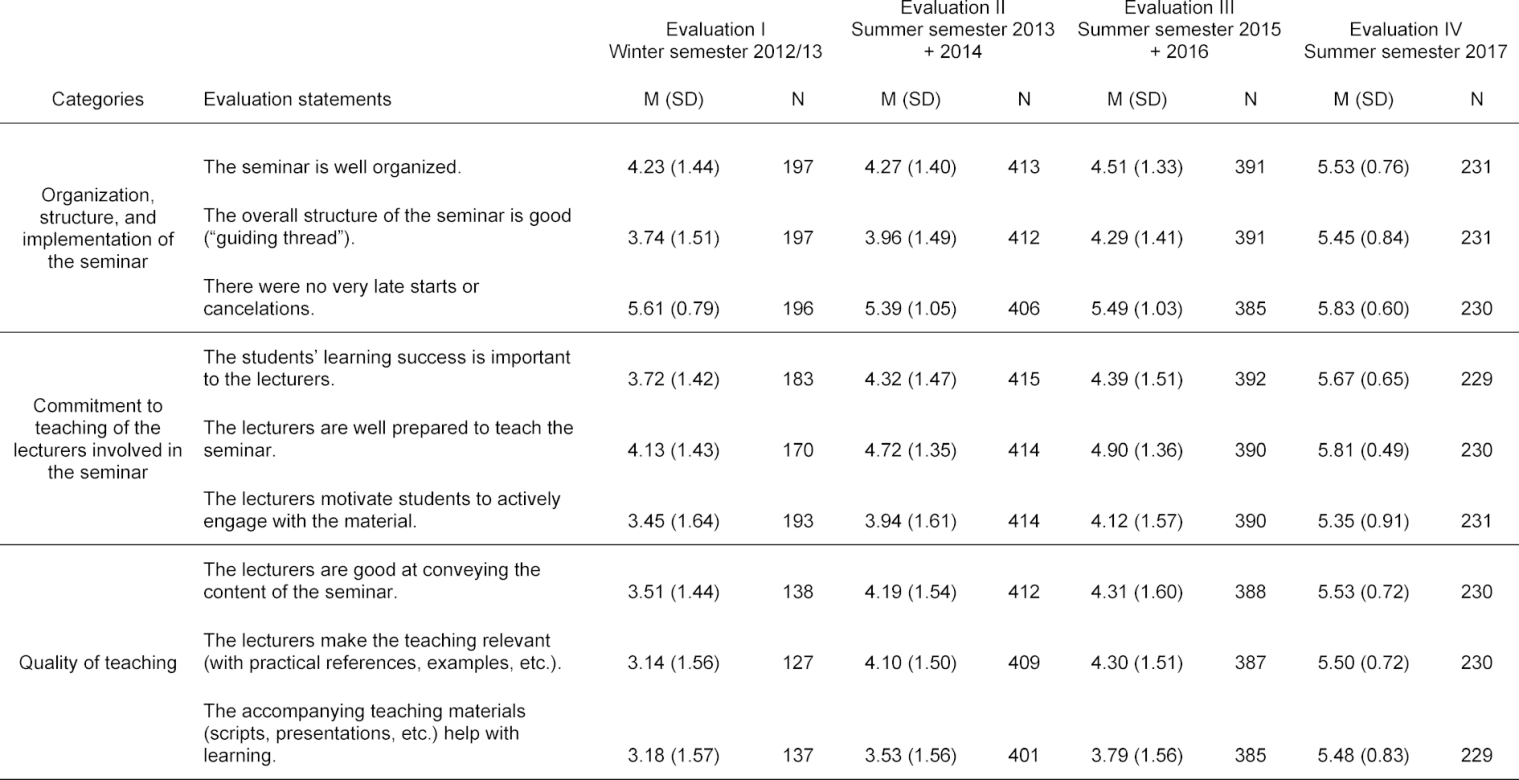
Statistical parameters of each evaluation statement that make up the scales for evaluating the seminar: “Organization, structure, and implementation of the seminar,” “Commitment to teaching of the lecturers involved in the seminar,” and “Quality of teaching.” The evaluation statements were assessed on a Likert-type scale ranging from 1=“does not apply at all” to 6=“fully applies.” Evaluation data from the pilot project 2016 were not included in the analyses.
