# Supplementary material for: Longitudinal curriculum development: gradual optimization of a biochemistry seminar
Source: GMS J Med Educ. 2019 Nov 15;36(6):Doc73. doi: 10.3205/zma001281 (PMC6905368; doi:10.3205/zma001281)
Supplement: Excerpts of critical free text (anonymized) [file JME-36-6-73-s-001.pdf]

1. Excerpts of critical free texts before the start of the longitudinal development process (winter semester 2012/13):

“In principle, rather publish a catalogue of learning goals for the biochemistry exams.”

"20 presentations in a row is simply not effective, you switch off at the latest after the 3rd or 4th presentation. Especially because hardly any of us were in the lecture, of course, and therefore we hardly have any prior knowledge. The whole thing ought to be arranged differently, unfortunately I also have no idea how."

"Students should have the opportunity to work through the learning content together in the seminar and not just have to listen to lectures by other students!"

"The students’ concentration had gone completely out the window at the latest after 5 presentations, the only one who was perhaps still present was the person speaking."

"[...] the timing of the seminar was very unfortunate. At the end of the really exhausting 3rd semester, it was just ‘get this over with, too ...’”

"It would be great if you would to work on cases like in anatomy: patient, symptoms, evaluation, diagnosis, what disease is it, treatment [...]"

"I am in favor of fewer or shorter presentations and for each one there is a short teaching unit by the lecturer on the topic, e.g. about current research, further thoughts or case studies."

"So much concentrated biochemistry in such a short time is overwhelming! You don’t remember much!"

"Overview and context are missing, unfortunately wrong time because at the end of the 3rd semester hardly anyone has any motivation left to work on the topic."

“It might be better to link the presentations to certain diseases, to make the relevance clearer and make the individual lectures more interesting for the listeners."

2. Excerpts of critical free texts after the first change/after the summer semester 2013:

"The point of the seminar is not really clear, especially because you have biochemistry again later anyway."

"The only drawback is the timing at the end of the semester, when everyone has ‘run out of steam’.”

"Why schedule it now? After the semester is ‘over’?”

"I think far too much time is estimated for this course. I don’t think it makes sense to schedule this seminar now or at least I don’t understand why it’s scheduled now. Maybe it should be moved to another semester [...]"

"Unfortunately, the theoretical foundation is missing sometimes because unfortunately the focus couldn’t yet be on biochemistry. Therefore, the timing of the seminar, which is actually very interesting, is somewhat unfortunate.”

After the summer semester 2014:

"In principle, I think the idea of distributing tasks to groups, which are then supposed to present them, is good, but some of the topics are somewhat complex so that in the end the lecturer gets stuck with the work anyway [...]."

"The seminar is interesting in principle, but waaaay too demanding for the current level of knowledge and then pointless parallel to the psych course because everyone's concentration is elsewhere anyway."

"The basic idea of the seminar seems to be very good, to clarify the relevance of the biochemistry topics in clinical practice. Unfortunately, the timing was not that good and not well chosen"

"In addition, there was also an exam during this period. Therefore, I don't think a seminar could be scheduled at a worse time".

"The timing was not good. Better, of course, than if it had been right before an exam. But how about scheduling the seminars during the semester, immediately after the first histology exam?"

3. Excerpts of critical free texts after the second change/after the summer semester 2015:

"The main problem of the seminar is that the students don't understand the content [...]"

"So far, the seminar I got the least out of. The tasks are not concrete, it is assumed that everyone has already dealt with the whole of biochemistry."

"The requirements were mainly too extensive for the short time, and the relevance of the content was not always clear."

"The topics are complicated, and it's difficult to concentrate for four hours. It would be better if students got the preparation questions earlier to work on them at home."

"Unfortunately, I think [...] the methodology and teaching principles [...] are not conducive to achieving learning success."

After the summer semester 2016:

"I attended the seminar extremely reluctantly because I could not see any relevance for exams or the like. This seminar wastes a lot of time that could be better spent working through other topics."

"The significance of the seminar and the connection to the lecture topics and the topics relevant for exams is not clear."

"The course should be more clearly structured."

"It would be better to limit the content to topics relevant for exams because what you learn is otherwise useless in terms of the exam and has been forgotten before it becomes relevant in the clinical part."

"Unfortunately, the lecturer did not manage to create a positive learning atmosphere at any of the sessions."

4. Excerpts of critical free texts after the third change/after the summer semester 2017:

"For the sake of accuracy, group tasks could have been checked or approved by the lecturer. Questions therefore arose afterwards about certain topics."

"Working on material in group work is not for everyone. Sometimes you learn better on your own."

"The seminar was dragged out a bit too much by lots of different group projects, so we often had trouble finishing on time."

"The topics were repeated too often. The role-playing games and lectures at the end did not help me to learn the subject matter better but cost me time that I could have used to study."

"Should moderate group work better — make more work materials available."

"I would have found it helpful if the spoken explanations in the educational films had also been available as text. That would have made it easier to study afterwards."

"I would have liked to work on more topics. The educational films were very detailed in some parts, but too brief in a few others."

"Smaller groups would be better."

"Criticism: At first I had trouble realizing what is really required and what I have to do (preparation, etc.)."

"What's the point of this seminar? The exams already demand enough, why presentations on top of them?"

Excerpts of positive free texts (anonymized)

1. Excerpts of positive free texts before the longitudinal development process (winter semester 2012/13):

"But in principle presentations are very well suited to getting a crash course"

"The requirements for the individual presentations were well formulated and clearly defined against the others."

"The topics in Module 6 could be easily presented in 8 min, so I learnt a lot more than in Module 4."

"The lecturer was very interested in the fact that we have a basic understanding and could contribute a bit to our presentations."
"The lecturer led the seminar very well and clearly explained things that were unclear!"
"The teacher was very friendly, nice."
"The lecturer complemented our presentations nicely with her slides, thank you!"
"I want to praise my group. Everyone really tried to give good presentations. Thank you!"

2. Excerpts of positive free texts after the first change/after the summer semester 2013:

"The lecturer spent a lot of time on the group's questions and any needs of individuals to focus on certain topics!"
"The lecturers made an effort to teach us as much knowledge as possible and took into account our lack of knowledge from the first semester."
"It was good that the lecturer tried to explain what we did not understand by using practical examples."
"I want to praise the lecturer, who explained the topics really very well and even answered the simplest questions. You didn't have to feel embarrassed if you didn't know something."
"I particularly liked the fact that the lecturer did not rattle off the whole module about proteins and biochemical facts, but really made sure that we fully understood the main material"

After the summer semester 2014:

"I like the direct link to illnesses."
"The short introductions to the diseases were very interesting"
"The lecturer explained the topics in a concise way and conveyed the relevant knowledge. Awesome, thank you"
"The topic was fascinating and very informative! Such practical examples / more detailed information can be gladly included more often!"
"A very interesting and informative course, where the basics of biochemistry could be repeated again and studied in more detail."

3. Excerpts of positive free texts after the second change/after the summer semester 2015:

"The seminar combines biochemistry with clinical practice and thus enables a better understanding. It also makes the subject more interesting."

"The clinical cases are very interesting, and you can learn a lot during the seminar."
"Find the use of current scientific texts really good."

"The lecturers also set their own priorities and mostly made sure they met the students at their level of knowledge and explained everything clearly."
"But in principle I think it’s good that there is a seminar on this topic because the topics themselves are fascinating."

After the summer semester 2016:

"Studying the direct consequences of small changes was interesting and showed how important correct biochemical processes in the organism ultimately are."
"In principle, an interesting seminar as regards the topics."
"However, by preparing for the course and studying afterwards, I learnt a lot of things that I could also use for the biochemistry exam."
"The topics and implementation of the seminar are done in a way that really arouses your interest, because biochemistry is very closely linked to pathology."
"The lecturer led it well and explained things very well when people asked questions."

4. Excerpts of positive free texts after the third change/after the summer semester 2017:

"All subjects should be structured in this way. My biggest problem — motivating myself to study — was simply non-existent in this seminar!"
"You remember everything that was discussed in the seminar and you only need to go through it again briefly before the exam — thanks to the great learning methods; instead of having to just memorize things (as is usually the case), the topics were discussed and put into a logical context, which the brain can remember better."
"Best seminar I have attended so far! I learned a lot from my own preparation with the educational films and really had the feeling that I spent my time in a meaningful way!"
"The Biochemistry Institute puts the most effort by far into optimizing ‘teaching’ and is very committed to doing so. One really has the feeling that the students’ learning success is close to the teachers’ hearts and that only biochemistry that is actually relevant for human medicine is tested."
"I found the format of the inverted classroom very successful, especially the educational films were really helpful in preparing for the exam. It’s nice when teachers make this much effort to explain the topics to the students."
"The seminar was very well structured and did a good job of covering the topics from the lectures in the first semester and refreshing them a bit. The educational videos were also helpful for studying and preparing for the exam."
"I like the idea behind the concept! It gets you back into biochemistry a bit, and it discusses topics that are very relevant for the exam and the later job!"

"The lecturer worked very hard and designed the seminar very well, showed an interest in us, but still challenged us and created a pleasant atmosphere — keep it up! :)"

"I really liked the idea of the inverted classroom. The preparation was more fun than usual or, rather, I actually did it."

"Inverted classroom — very helpful and promotes learning! I will definitely use the films in particular to prepare for the exam."
